# The Future of Laboratory Chemistry Learning and Teaching Must be Accessible

**DOI:** 10.1021/acs.jchemed.2c00328

**Published:** 2022-10-25

**Authors:** Orielia Egambaram, Kira Hilton, Jennifer Leigh, Robert Richardson, Julia Sarju, Anna Slater, Bethan Turner

**Affiliations:** † Department of Chemistry, University of Kent, Canterbury, Kent CT2 7NZ, U.K.; ‡ School of Social Policy, and Social Science Research, University of Kent, Canterbury, Kent CT2 7NZ, U.K.; § Department of Chemistry and Materials Innovation Factory, University of Liverpool, Liverpool L69 7ZD, U.K.; ∥ International Younger Chemists Network, https://www.iycnglobal.com; ⊥ Department of Chemistry, University of York, Heslington, North Yorkshire YO10 5DD, U.K.

**Keywords:** First-Year Undergraduate/General, Second-Year Undergraduate, Upper-Division Undergraduate, Graduate Education/Research, Laboratory Instruction, Safety/Hazards, Laboratory Management, Minorities in Chemistry, Student-Centered Learning

## Abstract

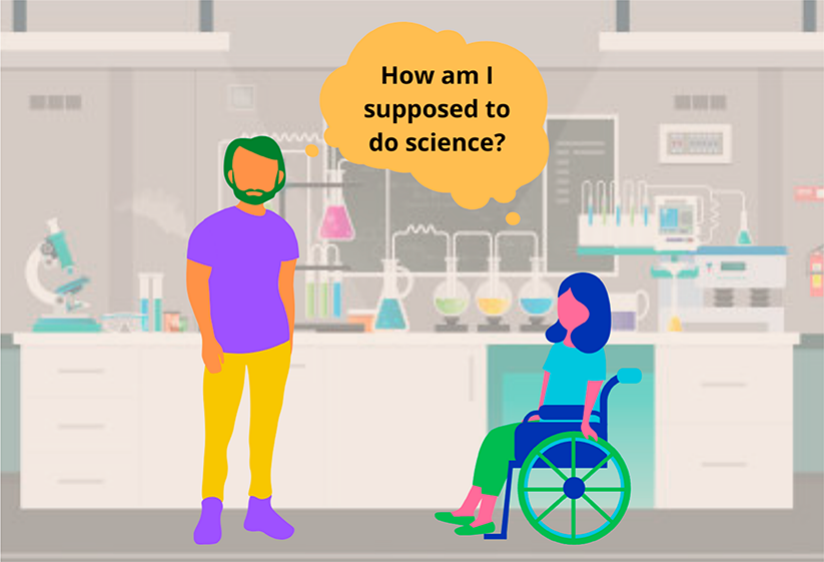

This commentary is a call to make the future of chemistry laboratories accessible and inclusive. We draw from research and lived experience to put forward a list of recommendations for laboratory-based teaching. Our authorial team includes undergraduate and postgraduate chemistry students, graduate teaching assistants, teaching-focused and traditional research and teaching academics, and a Diversity Equality Inclusion (DEI/EDI) academic expert. We all have lived experiences of disability, chronic illness, neurodivergence, and other marginalizations related to race, religion, sexuality, or other characteristics. We believe that laboratory-based chemistry learning environments, teaching, assessment, and resources should be accessible to all students and staff.

As a foundation to incorporate pedagogical approaches such as inquiry-based teaching into practical chemistry laboratories, we need to ensure that the environment itself is inclusive and accessible. There is a lack of diversity in science that urgently needs to be addressed.^1^ Women,^2^ Black,^3,4^ and disabled^5^ scientists are underrepresented in both education and careers as are other minority groups. In this commentary, we are specifically considering the impact of excluding disabled students and staff through the provision of laboratories that are inaccessible and call for all future conceptions of laboratories to build in inclusivity. However, we must note that disability, just like any other characteristic, is intersectional.^6^ Intersectionality means that the barriers experienced by a marginalized individual compound; that is, a disabled Black woman will face additional and unique challenges related to her disability, *and* her ethnicity, *and* her gender. Although our focus here is on disability and accessibility, we recognize that there is a need to address the lack of representation in chemistry more widely.

As authors, we all have lived experiences of disability, chronic illness, neurodivergence, and other marginalizations related to race, religion, sexuality, or other characteristics. Throughout, we use identity first language (disabled people not persons with disabilities) in adherence to the social model of disability, and conventions within the UK disability movement.^7,8^ For this paper, we conducted a collaborative autoethnography^9^ to research into and humanize^10^ our experiences of disability at different career stages, from undergraduate student to midcareer researcher. For this reason, we have chosen to write in the first person rather than in a passive voice. We also conducted online focus groups with 20 people from the UK, USA, Australia, and New Zealand talking about their experiences of disability and accessibility in the laboratory. This study had ethical approval from the University of Kent’s Centre for the Study of Higher Education. Each focus group lasted 1 h. The focus group participants included postgraduate researchers, postdoctoral researchers, early and midcareer researchers, university disability supporters, and health and safety officers.

## Defining Disability

**Figure 1:**
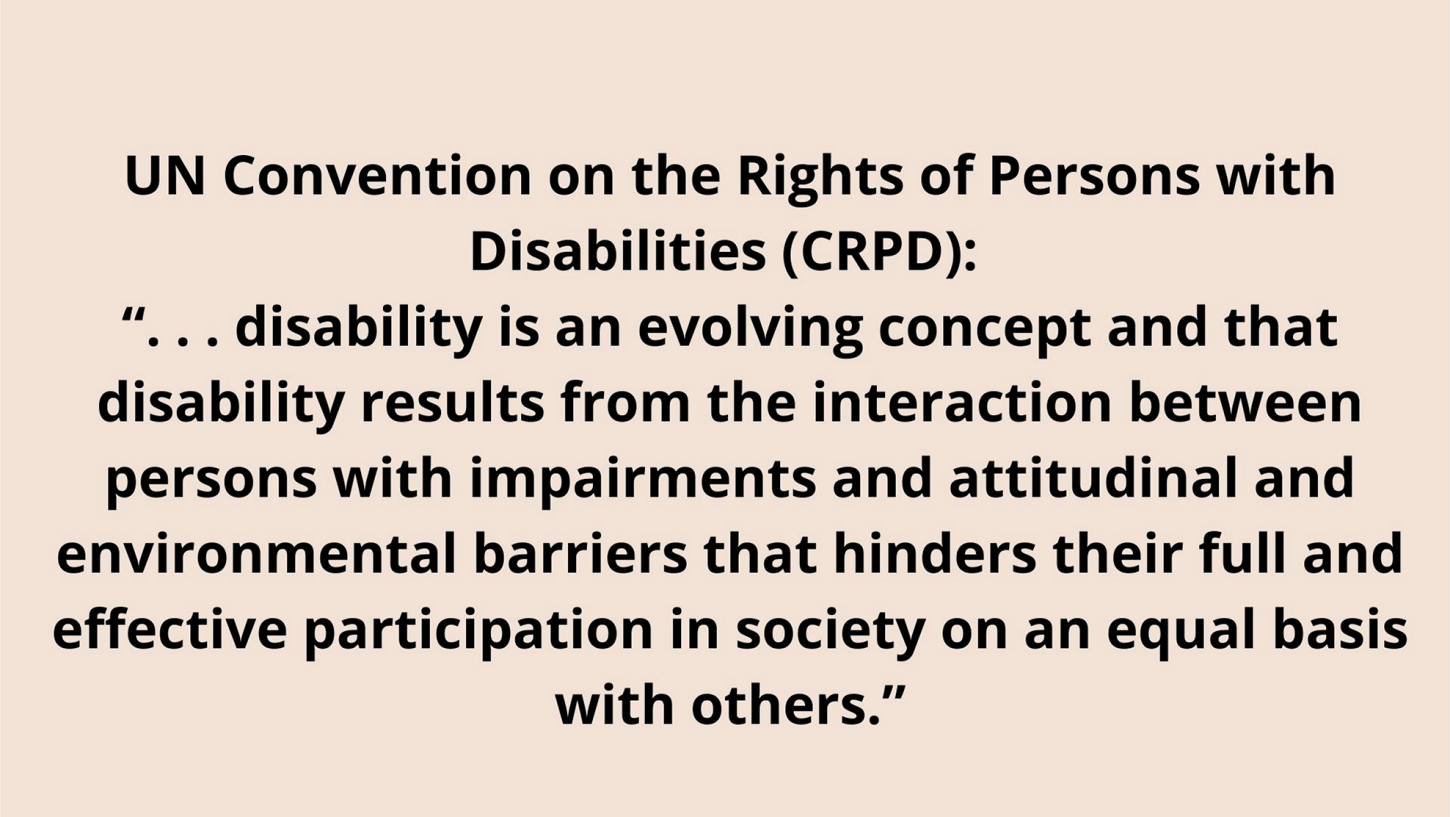
Quote from the UN Convention on the Rights of Persons with Disabilities (CRPD) adopted in 2006. [Descriptive text: text on pale pink background reads UN Convention on the Rights of Persons with Disabilities (CRPD): “...disability is an evolving concept and that disability results from the interaction between persons with impairments and attitudinal and environmental barriers that hinders their full and effective participation in society on an equal basis with others.”]

The definition of disability has been contested.^11^ The UK legal definition is that disabled people have a condition/s that severely affects their daily life, and that this condition/s has lasted or is expected to last for over 6 months. Disability can affect physical health, and/or mental health. Disability can be visible or invisible.^12^ Disability, and the impact of disability, can fluctuate.^13^ Disability can be something that an individual experiences from birth, or acquired at any age. Disability can be caused by many things, such as a chronic illness or condition, trauma, or an accident. The UN Convention on the Rights of Persons with Disabilities acknowledges that discriminatory attitudes and social inequalities are as disabling as an individual’s impairment (see Figure 1). In the UK, 33% of the population reports a chronic health condition or disability.^14^ This may be an underestimate, as not every person who would fall under the definition of disability chooses to disclose. Within the working population in the UK, 20% declare a disability.^14^ However, in academia, there is widespread underrepresentation of disabled staff;^15^ less than 4% of faculty staff disclose a chronic condition or disability.^16^ Proportions disclosing in Science, Technology, Engineering, and Mathematics (STEM) subjects, and other subjects with a pronounced gender imbalance in favor of cis-men, are even lower.^16^ This has implications for students as there is a lack of visible role models for disabled students and of lived experience of disability in decision making.

When it comes to students in the UK, the Royal Society Disability in STEM report states, “In 2018/19 the percentage of STEM first degree entrants with a known disability was 15.5% (33,530) compared to 16.4% (40,805) for non-STEM first degree entrants.”^5^ That report also recognizes that there is a disability awarding gap for first class/2:1 degrees: “STEM first degree qualifiers with a known disability achieved a lower percentage of “good honors” [than qualifiers who are not disabled]... The gap has narrowed slightly from 4.9% in 2007/08 to 4.4% in 2018/19. These percentage differences are statistically significant at the 95% confidence level. This trend is also observed for non-STEM subjects and the gap has narrowed from 3.6% in 2007/08 to 1.8% in 2018/19.”

These numbers demonstrate that there is a problem. We cannot tell from the numbers, however, whether disabled people are choosing not to study or work in science and chemistry, or whether they are there, and choosing not to disclose.^15^

### Disclosure

Inclusivity and accessibility in the laboratory should be integrated for all and not put in place only when an individual discloses. The decision to officially disclose a chronic condition or disability is an individual choice.^17^ Each person has to weigh up the perceived benefits and risks to them of disclosing within their context. Ableism, or the discrimination against disabled people, is endemic within academia.^18^ The risks of disclosure can be perceived to be higher if a condition is invisible, if it fluctuates, or if it has been stigmatized such as mental health or contested conditions such as fibromyalgia.^19^ Even if an individual chooses to disclose, they may not disclose the full extent that they are impacted.^20^ Disclosure has different implications for staff than for students, with the potential to affect grant success,^21^ progression,^22^ and exposure to discrimination and feelings of belonging.^23^ Both students and staff may be exposing themselves to microaggressions^24^ and work within inaccessible environments.^22,25^

### Experiences of Disability within Chemistry

**Figure 2:**
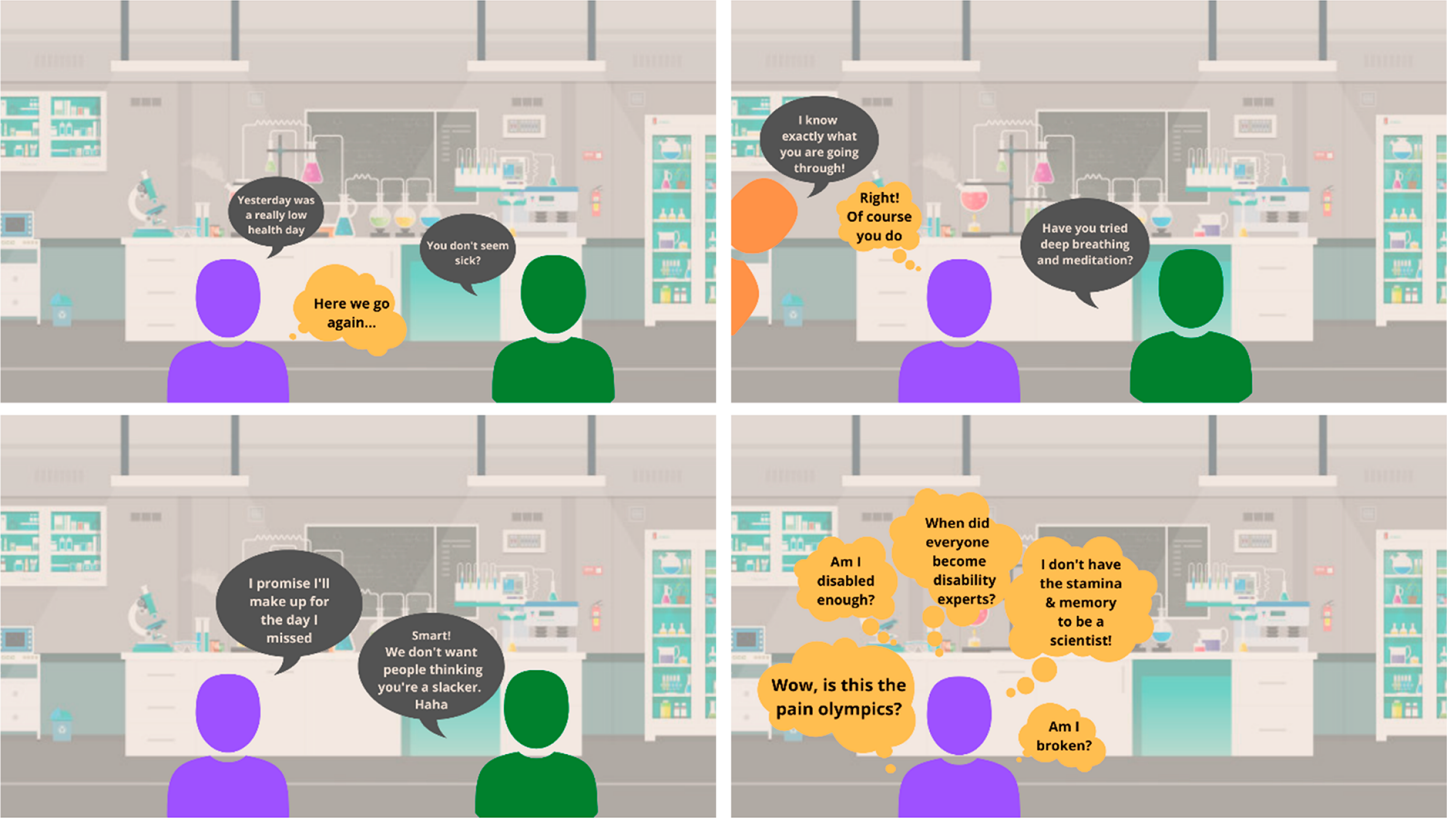
Cartoon representing toxic cultures experienced by disabled people in chemistry laboratories from other students, instructors, or colleagues. The purple figure represents a disabled person; the disability status or specific roles of the other figures is deliberately undefined. [Descriptive text: four panels showing cartoons of exchanges in the lab. Panel one: “Yesterday was a really bad day”, “You don’t seem sick”, thought bubble follows “Here we go again”. Panel two “I know exactly what you are going through”, thought bubble “Of course you do”, “Have you tried deep breathing and meditation”. Panel three: “I promise I’ll make up for the day I missed”, “Smart. We don’t want people thinking you are a slacker. Ha Ha!”. Panel four: thought bubbles “Wow Is this the pain Olympics?” “Am I disabled enough?” “When did everyone become disability experts?”, “I don’t have the stamina and memory to be a scientist” “Am I broken?”.]

If one is aiming to encourage more diversity in chemistry, it is vital that students and staff feel that they have a place in the discipline and that they belong.^26,27^ Without this, those who are marginalized are likely to experience a “chilly environment”^28^ and not see that they have the opportunity to succeed or progress.^22^ Inaccessible laboratories, or adjustments that are only brought in after disclosure, will emphasize the feeling of not belonging (see Figure 2).

### Inaccessible Chemistry Laboratories

Laboratory-based chemistry teaching is a core and significant component of undergraduate chemistry programs, through which students engage in the practice of science and develop scientific skills.^29,30^ It is highly valued by both students and instructors.^31^ However, experiences of laboratory-based teaching and the laboratories that they often take place in are not all positive or equitable. For example, with respect to neurodivergence, Flaherty highlighted that little consideration in chemistry education research and practice has been given to the impact of sensory overload experienced by many students and instructors in chemistry laboratories: “there seems to be little acknowledgment of just how difficult it can be for some to be in a room that is so noisy, bright, odorous and surrounded by hazards, risks, chemicals, glassware, electricity, gas, naked flames, eyewashes, body showers, fume hoods—the list goes on.”^32^ In the laboratory, sensory overload can be caused by sensory inputs with little or no informational content,^33^ such as noisy equipment, vibrations, bright and flickering lights, and chemical smells. Moving beyond sensory overload, Long and Kowalske reported that chemistry instructors “are not aware of the needs of D/HH [Deaf and Hard-of-Hearing] students, have limited experience with the D/HH community, feel unsupported in meeting the needs of D/HH students, and do not have or know how to access adequate resources to best support their D/HH students.”^34^ In terms of physical disability, our focus group participants spoke about how narrow or cluttered hallways and doors that were not automated made it more challenging for them to move around safely if they were using walking aids or wheelchairs. They came up with a number of recommendations we added to our list (see below), including allowing space for legs underneath benches or below fume hoods so people can work sitting on a stool or from a wheelchair, and having plug sockets that are accessible and not at the back of a deep bench. Experiences of disability are diverse; we need to listen to and learn from the experiences of disabled students and instructors to transform our instructional and assessment practices, resources, and environments.

### Lived Experiences of Inaccessibility in Chemistry Laboratories: A Collective and Anonymized Account

The following section is drawn from our collaborative autoethnography which focused on the impact of disability; here, we draw on personal experiences as well as interactions with other academics and students. As a group, we hold or have held roles including co-chair of Disabled Staff Networks, Departmental and School Equality Diversity and Inclusion (EDI/DEI) representatives and leads, departmental disability contact, and co-lead of the National Association of Disabled Staff Networks (NADSN) Science Technology, Engineering, Mathematics and Medicine Action Group.

Disabled students often get demoralized when the academic culture surrounding them holds a conventional wisdom that indicates they are unlikely to succeed, and if they do succeed, it means that it is unlikely that they are truly disabled and so should not have had accommodations. The dominant culture within chemistry seems to suggest that a “good chemist” can work long hours and know “all” the reactions. A disabled student may strive to reach these expectations at the expense of their health. In our collaborative autoethnography we shared examples of being challenged on individual adjustments, such as when a teacher questioned whether a disabled student could manage a STEM course due to their condition, and when a student on STEM course had been awarded extra time as an adjustment and this was then questioned by the invigilator or assessment facilitator. Having extra time can also cause issues with other students who perceive it as undeserved if they do not know why it has been awarded. This in turn puts more pressure on disabled students to disclose to their peers and who then feel that the topic of disability in general, and their disability in particular and how it impacts them and others and/or the adjustments they need, comes up every time they meet a new group of people. Questioning a student’s disability can lead to internalized ableism,^8^ which in turn leads to imposter syndrome and self-doubt. Numerous disabled students shared that the academic respect and intellectual image that others had of them was conditional, and that as soon as they showed a sign of struggle that respect was lost. This feeling of conditional academic respect also applies to other marginalized groups. These experiences breed a frustration that is exacerbated by comments from peers and faculty invalidating their experiences. For example, memorization of chemical knowledge is highly valued and respected whether in everyday conversations, in networks, or in exams where the main objective is to demonstrate memorization of synthetic routes, reagents, and equations. Neurodivergent and disabled students who have processing delay or brain fog can be at a disadvantage when it comes to displaying memorization of knowledge.^18^ However, it is important to note that even nondisabled students report forgetting information after a long summer holiday.

These attitudes seem to be very common and highlight the perception that sciences are a field where having a disability would mean you are likely to fail. Furthermore, if a student condition was well-managed and they performed well on their course, some classmates would argue that the student was “not really disabled”, leading them to start to question whether they really are disabled enough (internalized ableism)^13^ or if their accommodations provide an unfair advantage. Having these experiences can fuel self-doubts and cause imposter syndrome to be experienced in times when they are doing well.

Accommodations for disabled students are necessary. In our experiences, when disabled students start their chemistry degree there are generally very few such accommodations in place for laboratory work, and the onus is usually on the student to ask and/or push for them. Triggers and barriers for students were not accounted for and were often overlooked. However, for some students things are changing. Meetings reviewing the issues and how to resolve them can lead to vital improvements such as allowed rest breaks, informed demonstrators, and lab support supervisors appointed where necessary. These small changes significantly improved subject attainment and wellbeing. It also shows that the culture of the field can and should allow disabled students to succeed.

## Recommendations

Our recommendations include building in accessibility to future laboratories, just as good practice in education builds in inclusivity.^35^ This change will be achieved through a combination of physically accessible spaces, processes, policies, and attitudes. According to the health and safety experts we spoke to in our focus groups, many of these recommendations exemplify good practices in health and safety and good working practices: “a lot of things that are raised are not [only] accessibility, it’s about general health and safety and good practice” (health and safety officer). As such, these adjustments would make life in the lab better for everyone regardless of whether they had a disability, chronic illness, or neurodivergence. Our recommendations are grouped into three themes: physical adjustments, accessible resources, and changes that are needed within the culture of chemistry. While these recommendations will not rule out the need for individual accommodations, they will set a benchmark standard and include adjustments that will accommodate a wide range of disabilities, chronic illnesses, and neurodivergence. Furthermore, any processes for requesting and implementing individual adjustments must be inclusive for chronically ill people and those who do not identify as disabled.

### Physical Adjustments

The following table (Table 1) includes a range of adjustments or accommodations that could easily be retrofitted into existing laboratories, or built into the future laboratory, that would address the needs of many disabled students and staff.^36^ It is not an exhaustive list, and we focus on the moral duty for laboratories to be inclusive, rather than the legal duties of institutions which are beyond the scope of this paper. Building in accessibility would mean that each time an adjustment is required for an individual they would not have to go through a formal process asking for accommodations or “reasonable” adjustments. This is important to address the intersectional issues mentioned earlier; not everyone has equal access to diagnosis^37^ and the capacity to request accommodations. None of the adjustments suggested below would impair the functioning of a lab for those who are not disabled—to the contrary, they are likely to improve the lab experience for all.

**Table 1:** Summary of Inaccessible Lab Features, Who They May Impact Negatively, and Suggestions for Improvements Adapted with Permission from STEMEnabled^36^

Who Is It a Problem for?	Inaccessible Lab Feature	Laboratory Space Improvement
Visually Impaired	Dark cupboards	Install automatic lighting inside cupboards
	Dark workspaces	Ensure provision of portable and desk lamps
	Old wooden benchtops	Use matte white bench covers to aid visibility
Visually + Mobility Impaired	Cluttered walkways and trailing leads	Ensure clear access routes
Mobility Impaired	Steps and changes in level	Install ramps at all entrances and into rooms with a step/sill (ramps must have 1 in 12 gradients)
	Narrow walkways and doors	Ensure that access routes are free of clutter; these need to be at least 81 cm wide
	Push/Pull doors	Install automatic door openers to every door (not just main entrances to buildings)
	Sharp corners/high shelves reducing visibility	Fit convex mirrors in ceiling corners, and cover sharp corners
	Electrical switches difficult to reach	Use hanging plug sockets which can be moved as needed
	Taps difficult to reach or turn	Install lever action taps rather than twist action taps
	Fridges or cupboards difficult to open	Install handles on doors (not knobs or finger indents)
	Challenges opening and pouring or pipetting	Providing simple equipment to ease bottle/tube opening and stabilize pouring
	No space under the bench for a wheelchair user to pull in close, or a stool user to sit comfortably	Ensure at least 81 cm gaps are left in key places such as under sinks, under benches, and under fume hoods
	Chemicals stored on high shelfs or in very low cupboards	Store principal chemicals at between 100 and 150 cm height in bottles that are not excessively large
deaf/Deaf/Hard of hearing (d/D/HH)^a^	Solely audio alarms and signals	Install visual alarms such as flashing or red lights (that are LED-based and do not flicker) throughout the building
d/D/HH + Neurodivergence	Excessive machinery noise from sonicators and vacuum pumps	Move loud machinery to support rooms or laboratories, or seek to minimize its use.
		Regularly maintain equipment to prevent excessive noise.
	Loud radios and music	Minimize unnecessary noise where possible and allow the use of noise-canceling headphones (allowing for impact on buddy-safety)
Neurodivergence	Flickering lights	Upgrade from fluorescent lighting to LEDs to prevent flickering
	Fully open plan layout	Provide zoned or separate work spaces, delineating these by color/texture on the floor
	Cluttered work areas	Clear bench tops and have marked out areas for different activities
	Excessively colorful wall decor	Ensure that there are neutral and restful colors throughout

a “deaf” refers to anyone with a severe hearing problem; “Deaf” refers to people who have been deaf from before they could talk.

In addition, we recommend the provision of bench worktops of different heights or that can be adjusted in height; the provision of adjustable, comfortable stools for those who cannot stand for long periods; and the provision of fume hoods again with adjustable height worktops. Many of the adjustments we have recommended can be retrofitted at minimal cost such as visual alarms, and installation of handles for doors, automatic door openers, or lever taps. Some are more costly or could most easily be integrated into a laboratory at point of design such as fume hoods with space for legs/wheelchair users underneath them. Many of our focus group respondents spoke about difficulties in reaching stored chemicals or using pieces of equipment such as NMR instruments to run experiments. These challenges could be effectively ameliorated by employing experienced laboratory technicians and facilitators to provide chemicals to a bench in containers that are sized for easy use, and to set up experiments so that they can be run remotely.

### Accessible Resources

Building in accessibility requires considering the structure of the course, the lab manual or handbook, instructors notes, and any written materials to ensure that they are digitally accessible.^38,39^ Having accessible resources is ideally part of building in digital accessibility throughout the university. Digital accessibility ensures that these resources are accessible to anyone requiring or choosing to use a screen reader. Screen readers can be useful for anyone with a print disability, which might be due to a neurodivergence or specific learning disability such as dyslexia, or a physical disability that makes it challenging to hold or use a printed text.^40^ At the minimum on a course level, all course materials need to be available in advance and accessible to screen reader software available on a range of devices.

Further examples of the development of accessible resources in chemistry include building in flexibility and choice within the program,^39,41^ using 3-dimensional tactile models,^42,43^ applying computer vision to laboratory operations such as titrations,^44^ using software to describe chemical phenomena such as color changes,^45^ and using QR code labels and audio commentaries for commonly used chemistry laboratory apparatus.^46^

### Changing Culture

Changing the culture around accessibility and inclusion in chemistry is a long-term task, and it is one that should be undertaken by the whole community, including disabled chemists.^47^ Experiences of addressing other EDI concerns in chemistry suggest that embedding EDI expertise and taking a community-led approach are key.^48^ Due to the lack of space to discuss each point fully, we have compiled the following list as a starting point, and suggest the following:

- Institutions harness the wisdom of lived experiences by ensuring that diverse voices are involved in decision making at all levels, and encourage inclusive Student Partnerships.^49^
- Institutions provide disability inclusive specialized health and safety advice that is transparent, openly available, and used as support for students and staff rather than a means to exclude them from the laboratory. In doing this we would call on learned societies to provide reviews of specific recommendations such as that provided by the Committee for Chemists with Disabilities from the American Chemical Society.^50^ Such advice could be built on by also providing templates and examples of risk assessments for example.
- Institutions provide training around supporting disabled, chronically ill, or neurodivergent students, including training for instructors^51^ and teaching assistants.^52^ For example, concerning the use of assistance dogs in the laboratory,^53^ Ramp et al. called for inclusive guidelines for accommodating service dog handlers and raised concerns over “perpetuating negative bias” through the emphasis of “hazards without also emphasizing strategies for hazard minimization and the positive impacts of a more inclusive classroom”. In our focus groups, we heard from many neurodivergent students who had been forbidden to use noise-canceling headphones to prevent sensory overload. The Health and Safety experts we spoke to suggested that the use of such headphones would not prevent those students from working safely, but would inhibit them from being responsible for others’ safety unless they were working directly next to them, so that this should be factored into any “buddy” system for safe working.
- Laboratory courses and Health and Safety advice and training are proactively inclusive to the needs of most disabled laboratory demonstrators, (graduate) teaching assistants, and faculty in terms of design and materials. This would result in a more inclusive environment that is not dependent on individuals disclosing their needs. Individuals in need of specific adjustments must still be accommodated.
- There is a normalization of and encouragement for discussion of individual access needs. Reinholz and Ridgway argue that normalizing the discussion of access needs is a key step toward advancing disability justice.^54^ Instructors should check in with students and fellow instructors to ask whether their access needs are being met and create an environment where individuals can express their needs freely.
- Institutions encourage conversations and discussions that specifically aim to raise awareness and change and widen perspectives of what disability is to challenge outdated preconceptions. It is important to ensure that these discussions center lived experiences and support disabled teachers. This would include actively listening to and supporting student and staff networks such as NADSN, Women in Supramolecular Chemistry (WISC), TIGERSINSTEM, and DisabledInSTEM.
- Institutions encourage everyone involved with teaching to reflect on accessibility and inclusion of learning, teaching, and assessment. Institutions and individuals need to change and challenge the mantra of “if you can’t speed up, leave”.^46,55^ This will be achieved by ensuring that chemistry teachers reflect on their learning goals and assessments to be careful that they are not assessing hidden curricula, able-bodiedness, comfort in the learning and assessment environment, culture, belonging and dis-belonging to or in groups etc. instead of chemistry knowledge. For example; is there time pressure, do students with access to mentors and networks score higher, where is the required knowledge held and shared, is it in accessible forms? Are students able to develop and demonstrate their skills and knowledge while pacing themselves and taking care of their personal needs? Within laboratories, demonstrators and laboratory class leaders should encourage students to slow down and value students who are taking a thoughtful approach and making room for rest or their personal care needs such as bathroom breaks, eating, etc.
- Institutions ensure inclusive research experiences are included as course components, as laboratory teaching also includes teaching students how to research.
- Institutions ensure there is equal access to vacation bursaries/internships for disabled students.

## Conclusion

“There comes a point where we need to stop just pulling people out of the river. We need to go upstream and find out why they’re falling in.” Archbishop Desmond Tutu

We recommend an intersectional approach to inclusion and the design of the future laboratory of which accessibility is a core component. An intersectional approach is one which actively intends to address the combined barriers that individuals face due to intersecting aspects of their identity. To have the most impact, it is necessary to address inclusion regarding disability at the same time as also addressing inclusion regarding gender, race, ethnicity, or other marginalized characteristics within the wider environment. There is much work to be done if we are to achieve inclusive and accessible laboratory-based chemistry teaching for everyone. We have highlighted some of the obstacles and suggested directions for amelioration; we now invite you to reflect on our words and your experiences to identify actions you can take to transform laboratory-based chemistry learning and teaching, and evaluate the impact. While doing this, it is important to fully consult with staff and students to address intersectional barriers that go beyond the diversity of disability, chronic illness, and neurodivergence, and to employ the ideology “nothing about us without us”. As a first step, we suggest that every teaching laboratory immediately puts into place the list of physical adjustments laid out in Table 1 and utilize health and safety procedures effectively to create change. One of the health and safety experts we spoke to said, “for a lot of these issues there are fairly easy solutions. It’s not about removal of risk it’s about risk mitigation. We need to build labs with accessibility in mind.” Once teaching laboratories are embedding physical accessibility as the norm, this will be a catalyst toward open conversations and culture change.
